# The effects of angiotensin receptor neprilysin inhibition by sacubitril/valsartan on adipose tissue transcriptome and protein expression in obese hypertensive patients

**DOI:** 10.1038/s41598-018-22194-z

**Published:** 2018-03-02

**Authors:** R. Stinkens, B. W. van der Kolk, J. Jordan, T. Jax, S. Engeli, T. Heise, J. W. Jocken, M. May, C. Schindler, B. Havekes, N. Schaper, D. Albrecht, S. Kaiser, N. Hartmann, M. Letzkus, T. H. Langenickel, G. H. Goossens, E. E. Blaak

**Affiliations:** 1 Department of Human Biology, NUTRIM School of Nutrition and Translational Research in Metabolism, Maastricht University Medical Center+, Maastricht, The Netherlands; 2 Institute of Clinical Pharmacology, Hannover Medical School, Hannover, Germany; 3 Institute of Aerospace Medicine, German Aerospace Center (DLR), Cologne, Germany; 4 Profil GmbH, Neuss, Germany; 5 Clinical Research Center Hannover, Hannover Medical School, Hannover, Germany; 6 Department of Internal Medicine, Division of Endocrinology, Maastricht University Medical Center+, Maastricht, The Netherlands; 7 CARIM School for Cardiovascular Diseases, CAPHRI School for Public Health and Primary Care, Maastricht University Medical Center+, Maastricht, The Netherlands; 8 Translational Medicine, Novartis Institutes for Biomedical Research, Basel, Switzerland

## Abstract

Increased activation of the renin-angiotensin system is involved in the onset and progression of cardiometabolic diseases, while natriuretic peptides (NP) may exert protective effects. We have recently demonstrated that sacubitril/valsartan (LCZ696), a first-in-class angiotensin receptor neprilysin inhibitor, which blocks the angiotensin II type-1 receptor and augments natriuretic peptide levels, improved peripheral insulin sensitivity in obese hypertensive patients. Here, we investigated the effects of sacubitril/valsartan (400 mg QD) treatment for 8 weeks on the abdominal subcutaneous adipose tissue (AT) phenotype compared to the metabolically neutral comparator amlodipine (10 mg QD) in 70 obese hypertensive patients. Abdominal subcutaneous AT biopsies were collected before and after intervention to determine the AT transcriptome and expression of proteins involved in lipolysis, NP signaling and mitochondrial oxidative metabolism. Both sacubitril/valsartan and amlodipine treatment did not significantly induce AT transcriptional changes in pathways related to lipolysis, NP signaling and oxidative metabolism. Furthermore, protein expression of adipose triglyceride lipase (ATGL) (*P*_time*group_ = 0.195), hormone-sensitive lipase (HSL) (*P*_time*group_ = 0.458), HSL-ser660 phosphorylation (*P*_time*group_ = 0.340), NP receptor-A (NPRA) (*P*_time*group_ = 0.829) and OXPHOS complexes (*P*_time*group_ = 0.964) remained unchanged. In conclusion, sacubitril/valsartan treatment for 8 weeks did not alter the abdominal subcutaneous AT transcriptome and expression of proteins involved in lipolysis, NP signaling and oxidative metabolism in obese hypertensive patients.

## Introduction

Obesity is strongly associated with cardiometabolic risk factors^1^, which is reflected by an increased risk for arterial hypertension, heart failure and type 2 diabetes^2^. An impaired adipose tissue function and excessive fat mass in obesity represent key factors in the development of insulin resistance and related chronic diseases, including cardiovascular disease and type 2 diabetes^3^. Evidence suggests that impaired insulin sensitivity in obesity might be related to an altered renin-angiotensin system (RAS) and natriuretic peptide (NP) signaling in adipose tissue. Blockade of the RAS using angiotensin-converting enzyme (ACE) inhibitors or angiotensin type-1 receptor blockers (ARB) has been shown to improve insulin sensitivity and beta-cell function^4^ and reduces the incidence of type 2 diabetes^5^ as reviewed elsewhere^6^. However, results are not consistent^7^. In addition, NPs are positively associated with insulin sensitivity and low atrial natriuretic peptide (ANP) concentrations are associated with an increased risk of developing arterial hypertension and type 2 diabetes^8^. In accordance, neprilysin (NEP), which is involved in the degradation and inactivation of NP, is linked to insulin resistance, increased blood pressure and impaired lipid metabolism^9^. Therefore, combined RAS blockade and NEP inhibition might have synergistic beneficial effects on blood pressure and peripheral insulin sensitivity. We recently demonstrated that combined ARB and NEP inhibition, using sacubitril/valsartan (LCZ696), improved peripheral insulin sensitivity following 8 weeks of treatment compared to amlodipine (AMLO) in obese hypertensive patients^10^. However, the mechanisms underlying these beneficial effects remain to be established.

Evidence suggests that both the RAS and ANP affect adipose tissue metabolism, thereby determining insulin sensitivity^6,11^. It has been shown that valsartan (ARB) reduced adipocyte size, increased adipose tissue blood flow and decreased gene expression of angiogenesis, adipogenesis and macrophage infiltration markers^12^, which may have contributed to the valsartan-induced increased insulin sensitivity^4^. Furthermore, angiotensin II inhibited lipolysis *in vitro* in mature human adipocytes^13^, although conflicting findings on adipose tissue lipolysis *in vivo* in humans have been reported^14,15^. ANP has been shown to increase adipose tissue lipid mobilization and oxidation^11^ and we and others have recently demonstrated that ANP-mediated lipolysis is impaired in subcutaneous mature adipocytes from obese men with and without type 2 diabetes^16,17^.

Therefore, it is hypothesized that ARB and NEP inhibition with sacubitril/valsartan may affect adipose tissue function, thereby contributing to the observed improved peripheral insulin sensitivity in obese individuals^10^. The present study investigated the effects of sacubitril/valsartan compared to amlodipine treatment for 8 weeks on the abdominal subcutaneous adipose tissue transcriptome and protein expression profiles in obese hypertensive individuals.

## Methods

### Study design

Ninety-eight obese hypertensive patients participated in a multicenter, randomized, double-blind, parallel-group study to investigate the effects of sacubitril/valsartan (400 mg QD) compared with amlodipine (10 mg QD) treatment for 8 weeks. A detailed description of the study design, key inclusion and exclusion criteria of the patients and the primary results of this study have been described elsewhere^10^ (clinicaltrials.gov - NCT01631864, registered June 27, 2012). Briefly, the study included a screening period of up to 4 weeks, followed by a 4-week washout period and an 8-week randomized, double-blind and double-dummy treatment phase. Patients receiving antihypertensive medications at the time of screening discontinued the therapy during the washout period. During the treatment period, patients were randomized to receive either sacubitril/valsartan (400 mg QD) or amlodipine (10 mg QD) along with a matching placebo for 8 weeks. Patients were stratified into four groups based on the baseline Homeostasis Model Assessment of Insulin Resistance and statin use.

All patients gave written informed consent before participation and for the use of their adipose tissue biopsies in the current research investigation. The study was reviewed and approved by the Institutional Review Boards (IRB) of the Maastricht University Medical Center^+^ and at each participating centers’ IRBs. The study was performed in accordance with the Declaration of Helsinki.

Before and after treatment, abdominal subcutaneous adipose tissue biopsies were collected by needle aspiration under local anesthesia after an overnight fast. Subjects showing a treatment-induced increase in adipose tissue lipolysis *in vivo* (increase from baseline microdialysis glycerol concentration) following sacubitril/valsartan and subjects showing an unchanged lipolysis *in vivo* (change from baseline microdialysis glycerol concentration between −30 and +10 μmol/L) after amlodipine treatment^10^ were included in the following analyses. We determined adipose tissue gene expression profiles using microarray analysis in a subgroup of 70 patients who had a RNA Integrity Number (RIN) ≥5.0, as described in detail below. Also, in a subgroup (*n* = 12–13), we determined the expression of proteins involved in the lipolytic pathway, the natriuretic peptide signaling pathway and mitochondrial oxidative phosphorylation.

### Adipose tissue transcriptomics

After extraction, the integrity of abdominal subcutaneous adipose tissue total RNA was determined using the sample RNA Integrity Number (RIN), generated using an Agilent 2100 Bioanalyzer (Agilent Technologies Inc., Foster City, CA, USA). RIN values <5.0 indicated high level of sample total RNA degradation and were excluded from analyses^18^. Biopsies of 70 patients (*n* sacubitril/valsartan = 36, *n* amlodipine = 34) were eligible for transcriptional analyses (RIN ≥5.0).

Transcriptional data were generated using Affymetrix HG-U133plus2.0 oligonucleotide microarrays (Affymetrix Inc., Santa Clara, CA, USA) and the microarray files were pre-processed using the Robust Multi-array Average algorithm (RMA). The Chip Definition File (CDF) used for the RMA procedure corresponded to the public domain Michigan University Entrez CDF version 17.0. Transcripts showing median expression higher than 6 (log2-value) were combined and considered for statistical analyses.

All transcriptional analyses were performed in R: a language and environment for statistical computing (v.3.2.2 - R Foundation for Statistical Computing, Vienna, Austria. http://www.R-project.org). To identify and correct for potential sources of genomic data variation, the R/Bioconductor packages PVCA and ComBat (currently implemented as a function in the SVA R package) were used. To identify transcriptional changes induced by treatment, linear models followed by contrasts were defined and implemented using the LIMMA package (v.3.26.8). For this study, the threshold for statistical change significance was setup at nominal *P*-value ≤ 0.05. Nominal *P*-values were adjusted for multiple testing using the Bonferroni correction as well as the false discovery rate (FDR). A total of 8319 transcripts (out of assessed 18898 transcripts per microarray) fulfilled the expression level filtering criteria. The Bonferroni adjusted threshold was therefore defined as *P* < 6.01·10^−6^. The size of longitudinal treatment-induced transcriptional changes was expressed as Ratio Change from baseline [RC; (post-treatment level)·(pre-treatment level)^−1^)]. Thresholds for minimum relevant treatment-induced effects were defined as 0.66 ≥ RC ≥ 1.50 (i.e. RC range equivalent to absolute fold changes ≥1.5). The longitudinal treatment-induced effects on specific transcripts encoding for selected adipokines and gene products linked to the following biological processes: i) lipolysis, ii) fatty acid oxidation, iii) mitochondrial biogenesis, were also explored regardless of whether they surpassed or not the expression level filtering criteria stated above.

### Adipose tissue protein expression

A detailed description of the analysis can be found in the Supplementary Material. Briefly, abdominal subcutaneous adipose tissue was ground to a fine powder under liquid nitrogen and homogenized in RIPA buffer. The homogenate was lysed, vortexed and centrifuged and the supernatant was collected and stored at −80 °C. The protein concentration was determined by the Bradford-based protein assay. Next, solubilized proteins (15 µg) were separated on a precast gel and transferred onto a nitrocellulose membrane and quantitative Western Blot analysis was performed to determine the levels of proteins involved in the lipolytic pathway (adipose triglyceride lipase (ATGL), hormone sensitive lipase (HSL) and HSL serine 660 phosphorylation), the natriuretic peptide signaling pathway (natriuretic peptide receptor A (NPRA)) and mitochondrial oxidative phosphorylation (OXPHOS).

Protein expression data are expressed as mean ± S.E.M. All variables were checked for normal distribution by Shapiro-Wilk test and variables were Ln-transformed to satisfy conditions of normality (HSL, HSL S660 phosphorylation, NPRA and OXPHOS). Data was analyzed using two-way repeated measures ANOVA, with time (pre, post) and treatment (sacubitril/valsartan, amlodipine) as factors. Bonferroni post-hoc correction was applied when a significant time*treatment interaction was found. Calculations were performed using SPSS v.21 for Mac OSX (IBM, Chicago, IL, USA) and P ≤ 0.05 was considered statistically significant.

The datasets generated and/or analyzed during the current study can be made available by reasonable request to the corresponding author.

## Results

### Subject characteristics

Characteristics of patients contributing to the present study are shown in Table 1. Importantly, the two groups were well matched and there were no major differences in clinical characteristics between groups.

**Table 1:** Clinical characteristics of patients involved in transcriptomic and protein expression analyses.

**Transcriptomic analyses**						
	Sacubitril/valsartan *n* = 36		Amlodipine *n* = 34		Total *n* = 70	
Parameter	Baseline	Post intervention	Baseline	Post intervention	Baseline	Post intervention
Age (yrs.)	52.4 (8.56)	—	51.6 (9.21)	—	52.0 (8.83)	—
Gender (*n*)						
Male	28	—	25	—	53	—
Female	8	—	9	—	17	—
Weight (kg)	100 (18.2)	101 (19.0)	104 (15.8)	105 (16.4)	102 (17.1)	103 (17.8)
BMI (kg·(m^2^)^−1^)	32.4 (4.77)	32.6 (5.03)	33.6 (4.59)	33.8 (4.96)	33.0 (4.69)	33.2 (5.00)
Mean sitting SBP (mm Hg)	143.2 (13.26)	122.5 (9.97)	138.8 (11.61)	125.5 (12.28)	141.0 (12.60)	124.0 (11.17)
Mean sitting DBP (mm Hg)	89.6 (7.79)	81.9 (8.40)	90.4 (5.87)	83.4 (6.19)	90.0 (6.89)	82.6 (7.40)
**Protein expression analyses**						
	Sacubitril/valsartan *n* = 15		Amlodipine *n* = 12		Total *n* = 27	
Parameter	Baseline	Post intervention	Baseline	Post intervention	Baseline	Post intervention
Age (yrs.)	53.6 (6.92)	—	54.9 (7.04)	—	54.2 (6.87)	—
Gender (*n*)						
Male	13	—	8	—	21	—
Female	2	—	4	—	6	—
Weight (kg)	108 (19.2)	109 (19.8)	102 (14.4)	103 (15.1)	105 (17.1)	106 (17.8)
BMI (kg·(m^2^)^−1^)	34.3 (5.89)	34.7 (6.05)	33.1 (3.93)	33.6 (4.18)	33.8 (5.06)	34.2 (5.24)
Mean sitting SBP (mm Hg)	147.2 (10.62)	127.9 (11.72)	137.8 (12.59)	125.9 (13.37)	143.0 (12.27)	127.0 (12.27)
Mean sitting DBP (mm Hg)	92.0 (6.10)	68.3 (10.38)	87.5 (5.63)	66.7 (8.81)	90.0 (6.22)	67.6 (9.57)

Data are expressed as mean (SD).

### Transcriptomics

**Figure 1:**
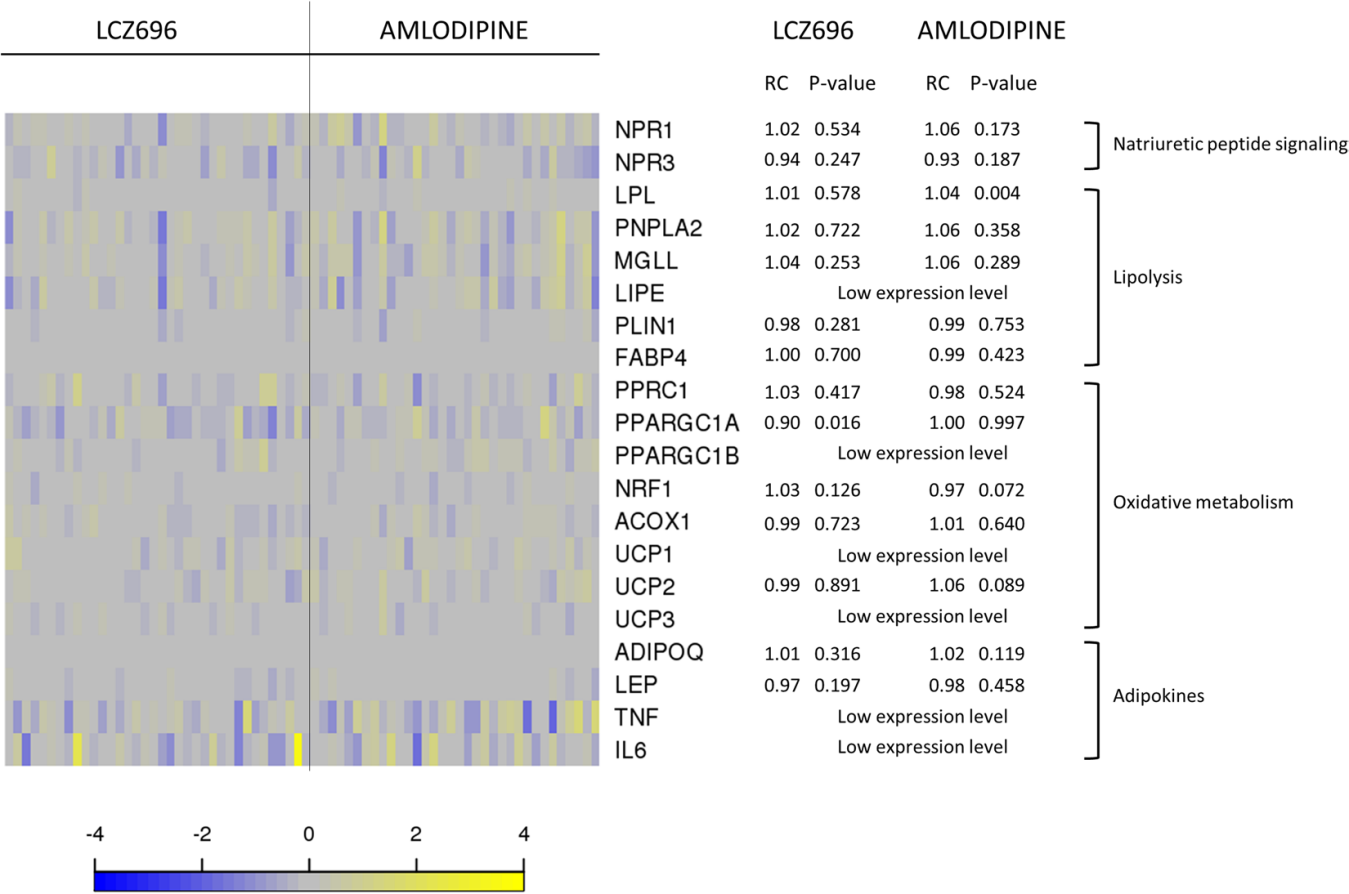
Targeted assessment of selected treatment-induced transcriptional changes in abdominal subcutaneous adipose tissue. The heatmap plot shows per patient longitudinal treatment-induced gene expression changes [defined as log2(post treatment expression) - log2(pre-treatment expression)] for selected transcripts related to natriuretic peptide signaling, lipolytic pathway, oxidative pathway and adipokines in abdominal subcutaneous adipose tissue (*n* = 70 patients; grouped per treatment arm). Color in the heatmap reflects transcript change from baseline per subject. Blue: downregulated transcripts; yellow: upregulated transcripts. The adjacent table depicts the corresponding transcript ratio change from baseline (RC) and *P*-value per treatment arm.

Although transcriptional assessments revealed 1443 longitudinally treatment-modulated transcripts (nominal *P* < 0.05), none of them fulfilled both thresholds for Bonferroni adjusted *P*-value and minimum treatment-induced RC effect. A set of selected transcripts is shown in Supplementary Table S1. Moreover, gene expression levels of transcripts involved in lipolysis, NP signaling, oxidative metabolism and adipokines (Fig. 1) showed no significant treatment-induced changes (defined based on the two thresholds stated above). Specifically, gene expression of ATGL (*PNPLA2*), MGL (*MGLL*), lipoprotein lipase (*LPL*), perilipin-1 (*PLIN1*) and fatty acid binding protein 4 (*FABP4*) was not significantly altered by sacubitril/valsartan treatment (Fig. 1). In addition, gene expression of natriuretic peptide receptors (*NPR1*, *NPR3*), adipokines (adiponectin (*ADIPOQ*), leptin (*LEP*)) and oxidative metabolism markers (peroxisome proliferator-activated receptor gamma co-activator-related 1 (*PPRC1*), peroxisome proliferator-activated receptor gamma co-activator 1 alpha (*PPARGC1A*), nuclear respiratory factor 1 (*NRF1*), acyl-coenzyme A oxidase 1 (*ACOX1*) and uncoupling protein 2 (*UCP2)*) were not significantly affected (Fig. 1). The FDR-adjusted *P*-values revealed only 28 transcripts in the sacubitril/valsartan group, in which only 1 transcript (Cholesteryl Ester Transfer Protein (CETP), Entrez Gene: 1071) fulfilled both the adjusted *P*-value and the absolute fold change criteria. In the amlodipine group, 15 transcripts showed an FDR-adjusted *P*-value ≤ 0.05 of which only 1 transcript (CETP) fulfilled both the adjusted *P*-value and the absolute fold change criteria. CETP was upregulated by both treatments.

### Protein expression

**Figure 2:**
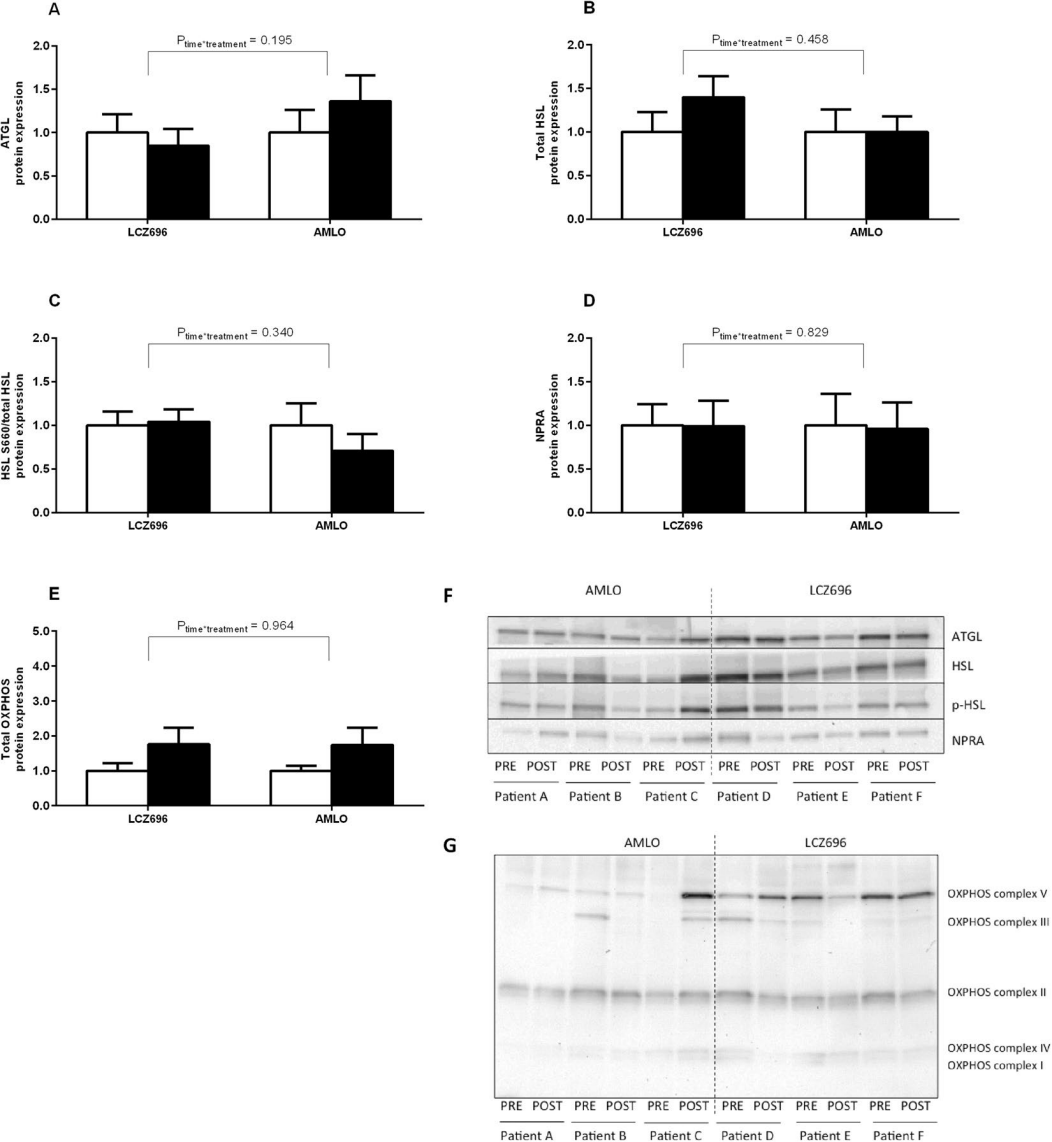
Protein expression in abdominal subcutaneous adipose tissue. Expression of proteins involved in the lipolytic pathway (ATGL, *n* = 12, (**A**); HSL, *n* = 13, (**B**); HSL serine 660 phosphorylation, *n* = 13, (**C**), the natriuretic peptide signaling pathway (NPR-A, *n* = 12, **D**) and mitochondrial oxidative metabolism (total OXPHOS, *n* = 13, **E**) expressed as fold change relative to baseline for each treatment arm (Sacubitril/valsartan: LCZ696 or Amlodipine: AMLO). Data are expressed as mean ± S.E.M. White bars: baseline values; black bars: post-treatment values. Representative (cropped) western blots of which membranes were probed with antibodies directed against total ATGL, total HSL, phosphorylated HSL (p-HSL) on Ser660 and NPRA (**F**) and uncropped western blots of OXPHOS protein expressions (**G**).

Sacubitril/valsartan (LCZ696) treatment did not significantly change protein expression of ATGL (LCZ696: 1.00 ± 0.21 vs. 0.85 ± 0.19 AU; AMLO: 1.00 ± 0.26 vs. 1.36 ± 0.30 AU; *P*_time_ = 0.583, *P*_time*treatment_ = 0.195; Fig. 2A), HSL (LCZ696: 1.00 ± 0.23 vs. 1.38 ± 0.24 AU; AMLO: 1.00 ± 0.26 vs. 1.00 ± 0.18 AU; *P*_time_ = 0.141, *P*_time*treatment_ = 0.458; Fig. 2B), HSL serine 660 phosphorylation (LCZ696: 1.00 ± 0.16 vs. 1.04 ± 0.14 AU; AMLO: 1.00 ± 0.25 vs. 0.71 ± 0.19 AU; *P*_time_ = 0.551, *P*_time*treatment_ = 0.340; Fig. 2C) or NPRA (LCZ696: 1.00 ± 0.24 vs. 0.99 ± 0.29 AU; AMLO: 1.00 ± 0.36 vs. 0.96 ± 0.30 AU; *P*_time_ = 0.775, *P*_time*treatment_ = 0.829; Fig. 2D). Furthermore, total OXPHOS protein expression (LCZ696: 1.00 ± 0.22 vs. 1.76 ± 0.48 AU; AMLO: 1.00 ± 0.15 vs. 1.74 ± 0.50 AU; *P*_time_ = 0.125, *P*_time*treatment_ = 0.964; Fig. 2E) remained unchanged following treatment. More specifically, OXPHOS complexes I-V were not affected (data not shown).

## Discussion

Here, we investigated the effects of sacubitril/valsartan versus amlodipine treatment for 8 weeks on the abdominal subcutaneous adipose tissue transcriptome and protein expression in obese hypertensive patients. We demonstrated that sacubitril/valsartan treatment did not significantly alter adipose tissue gene and protein expression of factors related to lipolysis, natriuretic peptide signaling and oxidative metabolism.

We recently demonstrated that in obese hypertensive patients sacubitril/valsartan treatment, which provides simultaneous ARB blockade and NEP inhibition, significantly increased peripheral insulin sensitivity^10^. Furthermore, sacubitril/valsartan slightly but significantly increased abdominal subcutaneous adipose tissue lipolysis, although no changes in whole-body lipolysis were observed^10^. Therefore, we hypothesized that RAS blockade and NEP inhibition has synergistic beneficial effects on abdominal subcutaneous adipose tissue metabolism and might underlie the observed improvement in insulin sensitivity. We showed that the abdominal adipose tissue phenotype was not significantly affected by 8 weeks of sacubitril/valsartan treatment in obese hypertensive patients. First, sacubitril/valsartan treatment did not elicit significant transcriptional changes in abdominal subcutaneous adipose tissue. In particular, no treatment-induced changes in expression of genes involved in lipolysis, the NP signaling pathway and mitochondrial oxidative pathway were detected. Secondly, the expression of proteins involved in these pathways, as well as post-translational modification of HSL, remained unchanged after the intervention.

Evidence suggests that both the RAS and NP system may affect adipose tissue metabolism, thereby contributing to improved insulin sensitivity^6,11^. It has previously been shown that angiotensin II decreased adipose tissue lipolysis *in vivo* in humans^14,19,20^ and in human isolated adipocytes^13^, which seems to be mediated via the angiotensin II type-1 receptor. However, increased adipose tissue lipolysis has also been reported^15^. In accordance with our results, long-term ARB treatment with valsartan improved insulin sensitivity in subjects with impaired glucose metabolism^4^, but adipose tissue gene and protein expression of several lipolytic enzymes remained unchanged^12^.

Several studies have shown that ANP promotes adipose tissue lipid mobilization and oxidation in healthy individuals^11^ via cGMP-mediated phosphorylation of HSL^21,22^. Furthermore, ANP induced mitochondrial biogenesis and uncoupling in human adipocytes from healthy, non-diabetic women^23^. Here, we did not find significant changes in gene and protein expression of markers involved in lipolysis, phosphorylation of HSL and mitochondrial oxidative metabolism, which may be explained by reduced ANP-mediated signaling in the study population. Indeed, an impaired ANP-mediated lipolysis has recently been described *in situ* in subcutaneous adipose tissue and *in vitro* in subcutaneous adipocytes from obese individuals^16,17^. Moreover, it has been shown that NPRC (NP clearance receptor) is increased in adipose tissue of obese hypertensive patients compared to lean and normotensive individuals^24^, together with increased NEP expression in obesity^9^. These data suggest reduced NP signaling and increased NP clearance in adipose tissue in obesity. This may explain the unaltered subcutaneous adipose tissue metabolic phenotype following sacubitril/valsartan treatment in the present study.

In general, the observed relative changes in gene expression were very modest, revealing only minor, potentially not physiologically relevant, changes in gene expression. Due to conservative multiple testing correction (Bonferroni correction), relevant treatment-induced changes in gene expression may have been missed. However, even when a less stringent method was used (FDR-adjusted data), only minor changes in gene expression were found.

Although we did not observe significant alterations in subcutaneous adipose tissue gene and protein expression of factors related to lipolysis, natriuretic peptide signaling and oxidative metabolism after 8 weeks of treatment with sacubitril/valsartan or amlodipine in obese hypertensive subjects, it remains to be investigated whether changes would occur in other adipose tissue depots, such as the visceral adipose tissue. Moreover, it would have been interesting to compare the effects of sacubitril/valsartan to placebo. However, since obese hypertensive patients are in need of anti-hypertensive treatment, we decided to use the metabolically neutral blood pressure lowering agent amlodipine as a comparator in this study instead.

## Conclusion

The present study demonstrated that simultaneous RAS blockade and NEP inhibition following sacubitril/valsartan treatment for 8 weeks did not significantly alter the adipose tissue metabolic phenotype in obese hypertensive patients. More specifically, abdominal subcutaneous adipose tissue gene and protein expression of factors involved in lipolysis, natriuretic peptide signaling and mitochondrial oxidative metabolism remained unchanged. Therefore, alterations in the adipose tissue metabolic phenotype do not seem to contribute to the improved peripheral insulin sensitivity following 8 weeks of sacubitril/valsartan treatment^10^.

## Electronic supplementary material

Supplementary Material
