# Supplementary material for: The effects of angiotensin receptor neprilysin inhibition by sacubitril/valsartan on adipose tissue transcriptome and protein expression in obese hypertensive patients
Source: Sci Rep. 2018 Mar 2;8:3933. doi: 10.1038/s41598-018-22194-z (PMC5834447; doi:10.1038/s41598-018-22194-z)
Supplement: Supplementary file 1 — Supplementary Material [file 41598_2018_22194_MOESM1_ESM.docx]

**The effects of angiotensin receptor neprilysin inhibition by sacubitril/valsartan on adipose tissue transcriptome and protein expression in obese hypertensive patients**

Stinkens R., van der Kolk B.W., Jordan J., Jax T., Engeli S., Heise T., Jocken J., May M., Schindler C., Havekes B., Schaper N., Albrecht D., Kaiser S., Hartmann N., Letzkus M., Langenickel T.H., Goossens G.H., Blaak E.E.

**Supplementary Material**

*Protein expression analysis*

Adipose tissue (~500 mg) was ground to a fine powder under liquid nitrogen and homogenized in radioimmunoprecipitation assay (RIPA) buffer (10 mM Tris (Calbiochem)-HCl (Merck, Darmstadt, Germany) buffered saline (Merck) with 0,1% SDS (Bio-Rad Laboratories Inc, Hercules, CA, USA), 1% Na-Deoxycholate (Sigma-Aldrich, St. Louis, MO, USA), 1% NP-40 (Fluka) and a protease/phosphatase inhibitor cocktail (Cell Signaling Technology, Beverly, MA, USA). The homogenate was lysed on iced and vortexed for 5 min and centrifuged at 20,000 g for 30 min at 10°C. The supernatant was carefully collected and aliquots were stored at -80°C. The protein concentration was determined by the Bradford-based protein assay (Santa Cruz Biotechnology, Dallas, TX, USA).

Next, solubilized proteins were separated on a precast gel (Criterion**™** TGX any kD, Bio-Rad Laboratories Inc, Hercules, CA, USA) and transferred onto a nitrocellulose membrane (Trans Blot**^®^** Turbo**™** transfer system; Bio-Rad). Differences in loading were adjusted to total adipose tissue protein content (via Ponceau S (Sigma-Aldrich, St. Louis, MO, USA) staining), and appropriate positive controls (lysates of abdominal subcutaneous adipose tissue) were included.

Thereafter, quantitative Western Blot analysis was performed to determine the levels of ATGL (Cell Signaling Technology, Beverly, MA, USA), HSL (kind gift from Prof. Cecilia Holm, [Department of Cell and Molecular Biology, Lund University, Sweden]), HSL serine 660 phosphorylation (Cell Signaling Technology, Beverly, MA, USA) and NPRA (Abcam, Cambridge, MA, USA). The secondary antibody was a horseradish peroxidase (HRP) swine-anti-rabbit antibody (DakoCytomation, Glostrup, Denmark). Furthermore, OXPHOS blots were probed with Total OXPHOS Antibody Cocktail (Abcam, Cambridge, MA, USA) and a secondary HRP-conjugated Rabbit-anti-Mouse antibody (DakoCytomation, Glostrup, Denmark). Antigen-antibody complexes were visualized using chemiluminescence by a ChemiDoc**™** XRS apparatus (Bio-Rad) and analyzed with Quantity One**^®^** software (Bio-Rad), which calculated the optical density units that are expressed as average intensity [average intensity = total intensity of the rows of pixels inside the band boundary divided by the number of rows, minus the background intensity].

**Table S1. Set of selected genes for the untargeted assessment of transcriptional changes in abdominal subcutaneous adipose tissue**

A total of 1443 transcripts were modulated by either sacubitril/valsartan (n=916) or amlodipine (n=668) treatment (nominal P≤0.05, no multiple correction applied). A set of selected genes with corresponding ratio changes from baseline (RC) and P-value per treatment arm is shown.

| **BIOLOGICAL PATHWAY** | **GENES ID** | **LCZ696 RC** | **LCZ696 P-value** | **AMLO RC** | **AMLO P-value** |
| --- | --- | --- | --- | --- | --- |
|  |  |  |  |  |  |
| INFLAMMATION | ALOX5AP | 0.76 | 0.001 | 0.93 | 0.483 |
|  | AOX1 | 0.93 | 0.083 | 0.89 | 0.025 |
|  | BLNK | 0.91 | 0.040 | 0.96 | 0.430 |
|  | BTK | 0.88 | 0.011 | 0.94 | 0.337 |
|  | C3AR1 | 0.90 | 0.072 | 0.88 | 0.024 |
|  | CCL16 | 1.14 | 0.009 | 1.01 | 0.838 |
|  | CCL18 | 1.24 | 0.040 | 0.97 | 0.781 |
|  | CD14 | 0.89 | 0.035 | 0.95 | 0.351 |
|  | CD163 | 0.94 | 0.222 | 0.91 | 0.027 |
|  | CD44 | 0.95 | 0.044 | 0.89 | 0.000 |
|  | CD74 | 0.98 | 0.530 | 0.94 | 0.028 |
|  | CLU | 0.96 | 0.036 | 0.98 | 0.372 |
|  | CTSS | 0.85 | 0.009 | 0.88 | 0.118 |
|  | CXCL9 | 1.20 | 0.042 | 1.15 | 0.134 |
|  | CYBB | 0.86 | 0.003 | 0.93 | 0.215 |
|  | E2F3 | 0.95 | 0.020 | 1.00 | 0.887 |
|  | FCER1G | 0.87 | 0.023 | 0.87 | 0.042 |
|  | FYB | 0.85 | 0.050 | 1.01 | 0.944 |
|  | FYN | 0.98 | 0.075 | 0.96 | 0.036 |
|  | GAB2 | 0.96 | 0.131 | 0.95 | 0.027 |
|  | GBP2 | 0.91 | 0.005 | 0.96 | 0.236 |
|  | HCLS1 | 0.90 | 0.022 | 0.98 | 0.687 |
|  | HLA-DMA | 0.99 | 0.660 | 0.93 | 0.033 |
|  | IFI30 | 0.89 | 0.153 | 0.77 | 0.000 |
|  | IFIT2 | 0.91 | 0.009 | 1.03 | 0.593 |
|  | IFNGR1 | 0.99 | 0.649 | 0.95 | 0.021 |
|  | IRF1 | 0.90 | 0.040 | 1.09 | 0.134 |
|  | ITGB2 | 0.85 | 0.031 | 0.82 | 0.020 |
| **BIOLOGICAL PATHWAY** | **GENES ID** | **LCZ696 RC** | **LCZ696 P-value** | **AMLO RC** | **AMLO P-value** |
|  | LCP1 | 0.85 | 0.016 | 0.82 | 0.019 |
|  | LYN | 0.87 | 0.009 | 0.98 | 0.823 |
|  | MAP2K1 | 1.03 | 0.048 | 1.04 | 0.119 |
|  | MAP2K4 | 0.94 | 0.004 | 0.98 | 0.414 |
|  | MAP3K7 | 0.97 | 0.044 | 0.93 | 0.004 |
|  | MAX | 0.96 | 0.041 | 0.98 | 0.514 |
|  | PAK2 | 0.97 | 0.023 | 0.99 | 0.447 |
|  | PIK3CB | 0.96 | 0.027 | 0.97 | 0.290 |
|  | PLA2G7 | 1.01 | 0.917 | 0.71 | 0.001 |
|  | PLCG1 | 1.07 | 0.010 | 0.99 | 0.748 |
|  | PPP3CC | 1.00 | 0.927 | 0.96 | 0.047 |
|  | PTPN6 | 0.88 | 0.042 | 0.98 | 0.779 |
|  | PTPRC | 0.83 | 0.017 | 1.00 | 0.989 |
|  | RAC2 | 0.85 | 0.034 | 0.96 | 0.652 |
|  | RIPK1 | 0.98 | 0.249 | 0.95 | 0.021 |
|  | STAT1 | 0.93 | 0.006 | 1.03 | 0.316 |
|  | SYK | 0.85 | 0.001 | 0.89 | 0.078 |
|  | TAB2 | 0.95 | 0.025 | 0.96 | 0.155 |
|  |  |  |  |  |  |
| OXIDATIVE METABOLISM | COX11 | 1.05 | 0.001 | 1.03 | 0.128 |
|  | COX4I2 | 1.09 | 0.016 | 1.11 | 0.024 |
|  | COX7A2 | 1.01 | 0.312 | 1.03 | 0.049 |
|  | COX7A2L | 0.97 | 0.028 | 0.98 | 0.288 |
|  | IDH2 | 1.02 | 0.530 | 1.10 | 0.011 |
|  | NDUFA5 | 1.01 | 0.831 | 1.06 | 0.049 |
|  | NDUFB4 | 1.03 | 0.179 | 0.94 | 0.047 |
|  | NDUFS1 | 1.00 | 0.939 | 1.08 | 0.010 |
|  | PDP1 | 0.94 | 0.002 | 0.99 | 0.497 |
|  | PPARGC1A | 0.89 | 0.016 | 1.00 | 0.997 |
|  | SLC25A11 | 1.01 | 0.804 | 1.10 | 0.009 |
|  | SLC25A16 | 0.99 | 0.820 | 1.10 | 0.019 |
|  | SLC25A18 | 1.08 | 0.041 | 1.07 | 0.117 |
|  | SLC25A27 | 0.99 | 0.862 | 0.92 | 0.046 |
|  | SLC25A33 | 1.04 | 0.337 | 1.10 | 0.038 |
|  | SUCLG1 | 0.96 | 0.017 | 1.02 | 0.417 |
|  |  |  |  |  |  |
| **BIOLOGICAL PATHWAY** | **GENES ID** | **LCZ696 RC** | **LCZ696 P-value** | **AMLO RC** | **AMLO P-value** |
|  |  |  |  |  |  |
| NATRIURETIC PEPTIDE | ADM | 0.95 | 0.027 | 0.95 | 0.071 |
| SIGNALING | EDN1 | 1.00 | 0.958 | 0.82 | 0.006 |
|  | FAP | 1.27 | 0.000 | 1.05 | 0.361 |
|  | GUCY1A2 | 1.12 | 0.000 | 1.11 | 0.001 |
|  | GUCY1A3 | 1.14 | 0.000 | 1.07 | 0.047 |
|  | GUCY1B3 | 1.12 | 0.000 | 1.04 | 0.150 |
|  | MME | 1.05 | 0.177 | 1.11 | 0.001 |
|  | PDE3A | 1.06 | 0.048 | 1.04 | 0.295 |
|  | PDE9A | 1.09 | 0.016 | 0.99 | 0.797 |
|  | RAPGEF5 | 1.09 | 0.031 | 1.04 | 0.362 |
|  |  |  |  |  |  |
| LIPID METABOLISM | ACACA | 1.06 | 0.153 | 1.14 | 0.008 |
|  | ADIPOR1 | 0.91 | 0.006 | 0.99 | 0.802 |
|  | ANG | 0.94 | 0.076 | 0.91 | 0.037 |
|  | CIDEA | 0.90 | 0.096 | 0.85 | 0.024 |
|  | ELOVL5 | 0.99 | 0.522 | 1.03 | 0.048 |
|  | ELOVL6 | 1.09 | 0.431 | 1.42 | 0.003 |
|  | ELOVL7 | 0.93 | 0.095 | 0.90 | 0.047 |
|  | GPAM | 1.01 | 0.498 | 1.08 | 0.002 |
|  | LPL | 1.01 | 0.578 | 1.04 | 0.004 |
|  | PPARGC1A | 1.08 | 0.016 | 0.93 | 0.997 |
|  | PLA2G4C | 0.89 | 0.043 | 1.00 | 0.096 |
|  |  |  |  |  |  |
